# Artificial Intelligence (AI) Trust Framework and Maturity Model: Applying an Entropy Lens to Improve Security, Privacy, and Ethical AI

**DOI:** 10.3390/e25101429

**Published:** 2023-10-09

**Authors:** Michael Mylrea, Nikki Robinson

**Affiliations:** 1 Department of Computer Science & Engineering, Institute of Data Science and Computing, University of Miami, Coral Gables, FL 33146, USA; 2 Department of Computer and Data Science, Capitol Technology University, Laurel, ME 20708, USA

**Keywords:** trustworthy AI, explainable AI (XAI), artificial general intelligence (AGI), entropy, information theory, autonomous human–machine teams and systems (A-HMT-S), cybersecurity, resilience, privacy, ethical AI

## Abstract

Recent advancements in artificial intelligence (AI) technology have raised concerns about the ethical, moral, and legal safeguards. There is a pressing need to improve metrics for assessing security and privacy of AI systems and to manage AI technology in a more ethical manner. To address these challenges, an AI Trust Framework and Maturity Model is proposed to enhance trust in the design and management of AI systems. Trust in AI involves an agreed-upon understanding between humans and machines about system performance. The framework utilizes an “entropy lens” to root the study in information theory and enhance transparency and trust in “black box” AI systems, which lack ethical guardrails. High entropy in AI systems can decrease human trust, particularly in uncertain and competitive environments. The research draws inspiration from entropy studies to improve trust and performance in autonomous human–machine teams and systems, including interconnected elements in hierarchical systems. Applying this lens to improve trust in AI also highlights new opportunities to optimize performance in teams. Two use cases are described to validate the AI framework’s ability to measure trust in the design and management of AI systems.

## 1. Introduction

This article develops an AI Trust Framework and Maturity Model (AI-TMM) to improve trust in in design and management of AI technologies. The framework distills down key ethical AI requirements from the literature. Then it applies a repeatable metrics of evaluation to assess and quantify how well key ethical AI characteristics are applied. Validation of the AI-TMM is conducted through two use cases. Key areas of exploration include ethical AI tradeoffs, the diversity of outputs, and predictability; security and explainability; and privacy and transparency. The study of structural entropy can help determine the right balance between performance, governance, and ethics in AI, especially in stochastic environments marked by randomness, disorder, and uncertainty. This research is timely as it fills gaps in the literature by answering critical questions, such as: What metrics of evaluation, equations, or models should be used to measure and determine the level of trust, control, and authority in AI systems? What are the metrics of evaluation and key performance indicators to measure and improve trust in AI? How can a maturity model methodology be applied to improve measurement of ethical AI in critical systems? What does this tell us about the security and privacy implications of popular AI applications? How can an entropy lens be applied to improve the design, deployment, and management of AI systems?

Conant’s (1976) entropy lens is applied to help answer these questions and establish a framework to improve design and governance of AI/ML systems [1,2]. This application assumes that knowledge is the absence of entropy production. Conant’s work focuses on biological and cognitive systems. Directly applying these concepts to AI systems on entropy production requires careful adaptation and consideration of the unique characteristics and challenges of artificial intelligence. For example, in developing trustworthy AI, this can be achieved by incorporating diverse training data, utilizing ensemble models, or implementing mechanisms for generating alternative responses. Thus, Conant’s work provides valuable insight into the study of complex systems, including the quantification of robustness and trust between interdependent parts in hierarchical systems. Conant (1976) noted that:

“Even when calculations are impossible, informal interpretations of informational equations shed interesting light on the behavior of systems. These informal interpretations are emphasized. It is shown that the requirements on a system for selection of appropriate information (and therefore blockage of irrelevant information), internal coordination of parts, and throughput are essentially additive and therefore compete for the computational resources of the system.”

Research on entropy applications to complex systems provides valuable insight into improving trust, robustness and resilience in the design and governance of AI/ML systems. The principle suggests that a system’s behavior can be predicted by maximizing the entropy of output subject to structural constraints. Thus, the most likely state of a system is the one that has the highest entropy output or the greatest amount of disorder, given certain structural constraints. In the context of AI systems, this principle highlights the importance of designing systems that can tolerate and adapt to unexpected changes and perturbations. We treat structure like knowledge. When a structure is optimized, its structural entropy production is minimal, allowing the maximum free energy to be allocated to maximizing a system’s output, maximum entropy production [3]. 

The proposed AI trust framework may help find a pareto optimum between interdependence and dependence of agents, privacy-preserving data and observability, diversity and predictability. Lawless [3] highlights that interdependence presents a measurement challenge linked to the coexistence of behavior and imagination, orthogonal aspects, and the ability to handle multiple tasks simultaneously. Applying this research to an AI Trust framework can help optimize moral and ethical guidelines in an A-HMT-S. This is timely, as lawmakers and citizens are increasingly concerned that we may have lost control of AI and it will soon control us. In establishing metrics for evaluating trust in AI, we can learn from research on entropy in complex systems and stochastic biological models where interdependent agents perform in complementary team roles (e.g., biological collectives, like ants [4] and plants or “mother trees” [5]). 

Trust in AI involves a social contract of assumptions between humans and machines on how a system or algorithm will perform [6]. Humans solidify assumptions and foster trust through consistency, reliability, and explainability in the systems they interact with. The framework applies an entropy lens to improve explainability and trust in General Artificial Intelligence (GAI) algorithms. The “black box” nature of generative AI lacks security and trust and creates entropy or disorder; the more disorder, the less trust and predictability there is in the work, organization, and/or team [7]. High entropy production, disorder, or randomness in AI systems can reduce human trust [8,9]. When AI outputs are unpredictable or unreliable, trust is lost; especially in A-HMT-S environments with high levels of uncertainty, conflict, and competition [3,10]. Lawless’ (2019) [3] research on entropy provides a valuable lens to help improve trust and performance of A-HMT-S. Conant’s (1976) work is also well adapted to study to improve measure of robustness and trust between interdependent parts in hierarchical systems [1]. 

First the article provides an overview of seven ethical areas that are critical to establishing trust. Each section provides an in-depth literature review of related research and distills down the various characteristics in tables. This enables users of the AI trust framework to apply various aspects of ethical AI based on their own goals for designing and managing AI. Next, the article provides a methodology for applying the AI trust framework and maturity model. The methodology is tested using two different illustrative use cases in companies. One company—the control use case—applies all of the seven areas to improve trust in the design and management of an AI system. The other use case highlights gaps in trust—security and productivity losses—for an illustrative company does not apply various critical aspects of trust defined by the framework. This article concludes by highlighting gaps that could be filled with future research to improve ethical AI guardrails with a focus on security, privacy and trust. 

## 2. Findings

### 2.1. Applying an Entropy Lens Highlights Opportunities for Improving Trust in AI

Entropy can help quantify the amount of uncertainty or randomness in an AI algorithm or system. It is often used to determine the efficiency of data compression algorithms or to measure the uncertainty of a random variable. In the context of entropy, an AI system with high entropy is one that has a large number of possible configurations or states, and it is more likely to be in a state that is highly disordered or unpredictable. Conversely, a system with low entropy has fewer possible configurations or states, and it is more likely to be in a state that is highly ordered or predictable. Entropy provides a value lens to help improve methods and analysis of trust in AI. Conant’s [2] work on entropy production, specifically the concept of “variety”, can also provide insights into improving trust in AI in complex systems. 

Applying this lens to large language models (LLMs) is especially important to improve contextual reasoning and use in a new era of GAI. In the context of AI, variety refers to the diversity and richness of responses or outputs generated by the system. High predictability in AI responses can also diminish trust as users may perceive the system as lacking intelligence, creativity, adaptability, and diversity required for contextual reasoning and advances in artificial general intelligence (AGI) [11]. Lawless’ (2019) [3] work noted how the lack of independence from government authorities had been a detriment to the market growth of China’s movie industry. How will the lack of output entropy and diversity in some LLMs lead to similar declines? How can improving transparency and XAI in the AI systems improve results? Conant’s (1976) work on entropy production, specifically the concept of “variety”, may help improve diversity and richness of responses generated by AI systems. Applying Conant’s entropy lens to improve trust in AI, the following approaches can be considered [1]:

Enhancing Response Diversity: By increasing the variety of responses generated by an AI system, it can demonstrate a better understanding of user inputs and offer more relevant and contextually appropriate outputs. This can be achieved through techniques such as incorporating diverse training data, utilizing ensemble models, or implementing mechanisms for generating alternative responses.

Reducing Predictability: High predictability (low entropy) in AI responses can diminish trust as users may perceive the system as lacking creativity or adaptability. By introducing controlled randomness or incorporating elements of surprise in AI outputs, it may make interactions with AI systems more engaging and less monotonous, but this should be explored.

Balancing Consistency and Novelty: While response diversity and unpredictability are important, striking a balance with consistency is also crucial. AI systems should avoid generating responses that are inappropriate, random, or inconsistent, as this can lead to confusion and mistrust. Finding the right balance between providing novel and diverse outputs while maintaining coherence and relevancy is key.

User-Centric Customization: Allowing users to customize AI systems according to their preferences and needs can improve trust. Providing options to adjust the level of response diversity or predictability can empower users to tailor their AI experiences, fostering a sense of control and personalization.

Explainability and Transparency: Trust in AI can be enhanced by providing causal explanations and insights upon request into how the system arrives at its responses. By offering visibility into the decision-making process and underlying algorithms, users can understand and evaluate the reliability and fairness of the AI system.

Conant’s work on entropy production primarily focuses on biological and cognitive systems. Directly applying these concepts to AI systems requires careful adaptation and consideration of the unique characteristics and challenges of artificial intelligence [1]. Applying an entropy lens from information theory, however, provides additional popular methods for analyzing the amount of information that is present in a system or transmitted between two systems. This is especially important as we want to improve trust in LLMs and GAI that lack transparency and explainability of the data used in both training as well as the weights applied to data outputs. To help explain what the lack of data transparency cannot, entropy can help measure the amount of missing information before reception. A couple of specific areas that can be adopted from the study of information theory and entropy include:

**Mutual information:** Quantifies the amount of information that is shared between two random variables. It is often used to determine the amount of dependence between two variables or to measure the amount of information that is transmitted between two systems [12]. 

*Opportunity to Improve Trust in AI*: Mutual information can be used to improve trust in AI by measuring the degree of association between the AI system’s output and the true underlying data. If the mutual information is high, it means that the AI system’s output is highly correlated with the true data, which can increase trust in the system’s ability to accurately predict outcomes. Additionally, mutual information can be used to identify and quantify any biases in the training data, which can further increase trust in the AI system by ensuring that it is not making predictions based on biased information [13].

**Kullback–Leibler Divergence (KLD):** Quantifies the difference between two probability distributions. It is often used to compare the accuracy of statistical models or to measure the amount of information that is lost when approximating one distribution with another [14]. 

*Opportunity to Improve Trust in AI:* Presupposing the existence of a true distribution that is to be learned, KLD can be leveraged to improve trust in the model by comparing the predicted probability distribution to the true distribution. For example, if the KLD is small, it suggests the model’s predictions are like the true distribution, indicating that the model is likely to be trustworthy. For example, in reinforcement learning, KLD is used as a regularization term in the objective function. This encourages the model’s predicted action distribution to be similar to the true action distribution, which can increase trust in the model’s predictions. Similarly, in Generative models, where KLD is used to measure the similarity between the generated and real data distribution, a small KL divergence suggests that the generated data are similar to the real data, indicating that the model is likely to be trustworthy. In general, using KLD as a measure of trust in AI can help identify when a model’s predictions deviate significantly from the true distribution, which can help identify when the model is not performing well and may need to be adjusted or improved.

**Channel capacity:** Determines the maximum rate at which information can be transmitted over a communication channel with a given level of error. It is often used to design communication systems or to analyze the performance of existing systems [15].

*Opportunity to Improve Trust in AI:* Channel capacity can be used to improve trust in AI by ensuring that the system is able to process and transmit large amounts of data quickly and accurately. A higher channel capacity enables an AI system to process more data, which can lead to more accurate predictions and better performance overall. Improving channel capacity enables an AI system to process data faster. This can help improve performance and even trust in A-HMT-S; especially with time-sensitive applications such as autonomous driving or financial trading. Furthermore, channel capacity can be used to verify the integrity of data being transmitted between the AI system and other devices or networks, by using error correction codes. This can increase trust in the AI system by ensuring that the data being used for predictions are not corrupted or tampered with during transmission. While channel capacity can help to improve trust in AI by allowing it to process more data accurately and quickly, there are several potential limitations when privacy preserving machine learning (PPML) is applied. Future research should explore those challenges and potential limitations through an entropy lens. 

Entropy represents the unavailability of a system’s free energy to do work. Entropy is the disorder in a system; the more disorder, the less work that it can perform. Second, the more disorder, the less predictable an organization becomes. Applying entropy research can provide a valuable lens to improve trust and resilience in the design and management of AI systems [2]. When designing AI systems, it is important to consider the range of possible configurations or states that the system might encounter. For example, autonomous vehicle systems encounter a wide range of weather and traffic conditions, each of which presents a different set of potential states or configurations. Similarly, in the case of a healthcare AI system, the system might encounter a wide range of patient populations and medical imaging technologies, each of which presents a different set of potential states or configurations. Designing AI systems that are trustworthy and resilient requires maximizing entropy subject to constraints. This requires designing systems that can tolerate and adapt to a wide range of possible states or configurations, while also ensuring that the system remains within certain constraints or boundaries. For example, in the case of an autonomous vehicle system, the system must be able to adapt to a wide range of weather and traffic conditions, while also ensuring that it stays within the boundaries of the road and obeys traffic laws. To achieve this, AI systems must be designed with flexibility and adaptability by designing algorithms that can detect and respond to unexpected inputs or changes in the environment and by building in redundancy to ensure that the system can continue to operate even if one component fails [16].

### 2.2. AI Trust Framework and Maturity Model (AI-TMM)

Designing AI systems that are trustworthy and resilient also requires improved metrics of evaluation. In realization of that goal, the AI Trust Framework and Maturity Model (AI-TMM) employs a maturity model approach to construct measurements for assessing the security of AI across its design and implementation stages. AI and machine learning play crucial roles in enhancing the capabilities and efficiency of complex systems. AI can optimize structural entropy by utilizing algorithms and techniques that enhance the organization, predictability, and efficiency of complex systems. However, if the inputs for AI modeling, training, and learning are compromised, the outcome of a high-fidelity, real-time portrayal of a physical entity will be adversely affected, jeopardizing the integrity and potential availability of the system or processes. This could lead to corrupted simulations and analyses, generating erroneous scenarios, behaviors, and other distorted signals for operators and end users. Instead of bolstering and optimizing the capabilities and effectiveness of AI systems, attacks or compromises of data lineage and AI access controls can impact the integrity and reliability of systems. This, in turn, could result in misinterpretation, manipulation, and deterioration of trust between autonomous human–machine teams and systems. 

The AI-TMM (AI Trust Maturity Model) offers a flexible and adaptable framework to seamlessly integrate organizational needs related to security, governance, risk, and compliance. For instance, if a customer using Google Cloud Platform (GCP) adopts Google’s Secure AI Framework (SAIF) to address security risks in their AI system, a well-structured security framework should facilitate the application and execution of associated controls. Similarly, if an end user is incorporating governance frameworks like the NIST Cybersecurity Framework, an effective framework should provide a user-friendly methodology for integration. Restricting the adaptability of a framework hinders its adoption, sustainability, and implementation, potentially leading to a false sense of security.

The AI-TMM employs a maturity model approach to gauge levels of maturity indicators in selected controls. AI-TMM’s Maturity Indicator Levels (MILs) are briefly outlined as:

- Fully Implemented (MIL Score of 3): Control is Tested, Managed, and Documented
- Largely Implemented (MIL Score of 2): Control is Managed and Documented
- Partially Implemented (MIL Score of 1): Control is Documented
- No control (MIL Score of 0): No Control is Documented, Managed, or Tested

These MILs are independently applied to each principal domain, enabling users to operate at different MIL ratings across domains. Organizations might operate at MIL2 in one domain, MIL3 in another, and MIL0 in yet another. Within each domain, the MILs are cumulative, requiring fulfillment of all practices within the specified level and its preceding levels. For example, reaching MIL2 in a domain necessitates completing all practices in MIL1 and MIL2. Similarly, achieving MIL3 requires completing practices in MIL1, MIL2, and MIL3. Enhancing the maturity level of crucial controls can heighten trust and security for A-HMT-S. However, optimal MIL levels will differ among organizations due to resource diversity, goals, and potential business impacts in case of exploitation.

These metrics can enhance transparency in decision-making processes, facilitate clear communication channels, shared decision-making, effective collaboration, and a shared sense of responsibility and accountability. This is valuable in the context of AI systems, where a neural network with high structural entropy might have randomly connected layers and nodes, potentially leading to unpredictable behavior. Similarly, a neural network with low structural entropy may have a well-defined architecture, but still requires ethical guardrails in its design and management for it to be a secure, safe, and sustainable solution.

**Figure 1:**
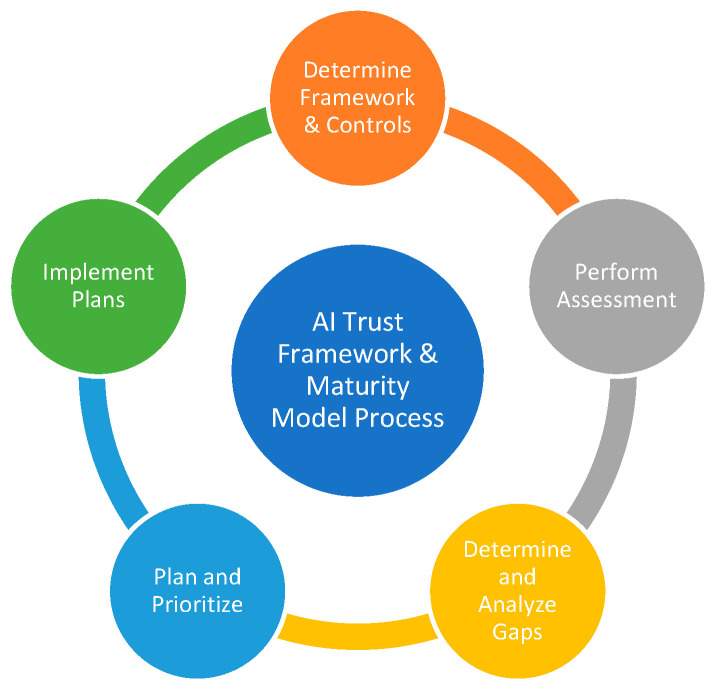
AI-TMM methodology (Mylrea 2023) [17].

Applying the AI-TMM methodology involves the steps summarized in Figure 1. 

**Figure 2:**
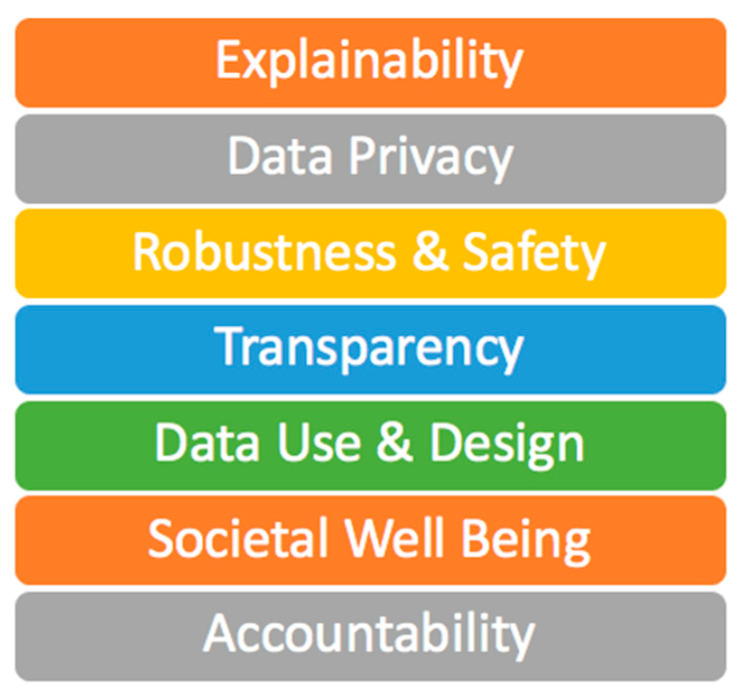
AI trust framework key pillars.

Step 1: Determine Governing Frameworks and Controls: Depending on the resources available to perform the evaluation and the goals of the organization, a subset of controls from the 7 trust pillars (see Figure 2) can be evaluated. For example, if an organization has limited resources and wants to focus more on privacy concerns than transparency, the modular design of AI-TMM will facilitate that goal.

Step 2: Perform Assessment: Evaluate the desired framework controls using the maturity indicator level methodology.

Step 3: Determine and Analyze Gaps: Evaluate the identified gaps through the lens of organizational objectives, resources, and potential consequences if these gaps or vulnerabilities are exploited.

Step 4: Plan and Prioritize: Compile a list of gaps and potential consequences while acknowledging organizational limitations. If specific business impacts or risks are deemed unacceptable, prioritization and strategic resource allocation are essential to mitigate associated risks. Conducting a cost–benefit analysis on proposed actions and priorities is necessary once strategies are identified to address the gaps.

Step 5: Implement Plans: By applying AI-TMM, the evaluation metrics may enable more efficient resource allocation to manage risks in a measurable and consistent manner. 

As security and trust in AI evolve, it is important to adapt how we measure and evaluate related metrics in the design and management of AI. Following the release of OpenAI’s GPT-4, a letter was signed by over 1000 researchers and technologists, urging for a temporary halt of six months in AI development as it presents “profound risks to society and humanity [18]”. As AI technologies improve our insight, inference, and predictability in big data sets, human teams will become increasingly reliant on these gains in autonomy and efficiency. For these gains to be ethical and sustainable, however, we need to improve the quantification of trust (e.g., explainability, transparency, auditability). Improving AI trust and ethics requires improved metrics of evaluation to measure the social contract and relationship between human–machine teams. This approach will help improve contextual awareness and predictability in an ethical and sustainable way for AI users [17]. 

The following research outlines critical characteristics of trust—both theoretical and applied—to help improve trust in AI systems and technology. Key areas of focus include the key trust pillars of the AI Trust Framework (Figure 2): i. Explainability (XAI), ii. Data privacy, iii. Robustness and Safety, iv. Transparency, v. Data Use and Design, vi. Societal Well-Being, vii. Accountability. A maturity model methodology is applied to improve the metrics of evaluation of each of these pillars in the design and management of AI systems. 

Each item in this list fills a critical pillar underpinning the AI Trust Framework; however, this should not be considered an exhaustive list. Assumptions—like trust—are not static and change as our reality changes. Large language models (LLMs) and generative AI can help improve human knowledge and learning by finding inference and insight in large data sets, but we cannot assume that the objective function, parameters, and weights that govern AI outputs are optimized to govern complex systems (e.g., people, biology, cyber weapons). Entropy production may help improve an AI model’s insight into the stability, equilibrium, and dynamics of complex systems by understanding how they respond to changes and perturbations. For example, Generative Adversarial Networks (GANs) indirectly involve the concept of entropy production in combining a generator and a discriminator in neural networks. The generator attempts to create data instances that resemble real data, while the discriminator tries to distinguish between real and generated data. The two networks are trained in a competitive manner, with the generator improving its ability to generate realistic data as the discriminator becomes better at differentiating between real and generated data. For the model to reach equilibrium, the generator minimizes the difference between the entropy of the generated data distribution and the entropy of the real data distribution [19]. An entropy lens may help us push the envelope in understanding the metrics of evaluation, opportunities, and costs, as we push the innovation envelope on these systems. 

The following sections explore the key pillars of the AI Trust Framework.

#### 2.2.1. Explainability (XAI)

Explainability, or Explainable Artificial Intelligence (XAI), is imperative for maintaining ethical standards in AI by fostering transparency, accountability, and trustworthiness [20]. XAI (see Table 1) pertains to the capability of AI systems to offer explanations that are comprehensible and can be interpreted. This allows stakeholders, such as users, regulators, and those impacted, to understand and assess the rationale behind the decisions and actions taken by AI systems [21]. Explainability plays a critical role in identifying and rectifying biases, ensuring fairness, and detecting any potential errors or unintended consequences [22]. By promoting transparency and facilitating effective human oversight, explainability contributes to the ethical and responsible utilization of AI systems. Explainability (XAI) plays a critical role in the AI Trust Framework. Table 1 explores methods and analysis that improve explainability in AI. There are several methods and techniques that can be used to improve the explainability of AI models, including: 

**Feature Importance Analysis** to determine which input features are most important for making a prediction.

**LIME (Local Interpretable Model-Agnostic Explanations)** to generate explanations for individual predictions by approximating the model locally with an interpretable model [23]. LIME can be applied in finance to understand a model’s local decision-making process, such as explaining why a credit card application was rejected: by generating a synthetic dataset from the application, obtaining predictions for perturbed instances using the model, fitting an interpretable model to the synthetic data, assigning instance weights, and using this to explain the rejection through feature importance from the interpretable model.

**Shapley Additive Explanations** applies a cooperative game-theoretic approach to explain the output of ML models [24]. 

**Attention Visualization** to visualize the attention mechanism to understand how the model is making decisions.

**Counterfactual Analysis** to generate examples of inputs that would cause the model to make a different prediction [25]. 

**Model Distillation** to leverage smaller AI models to mimic the behavior of more complex models to facilitate explainability, and explainable AI (XAI) frameworks that facilitate model interpretability.

**Table 1:** Explainability (XAI) documentation and metrics of evaluation in the AI-TMM.

Key AI Ethical Requirements—Trust Factors and Assumptions	Documentation	Metric of Evaluation
**Trust Factors and Assumptions** AI/MI algorithms produce results that are repeatable, interpretable, intuitive, and human-understandable explanations. “Interpretability as a technical term focusing on the clarity of the system’s internal logic and explainability as the ability of a human user to understand that logic.” [26]	Quantification of accuracy of results that are repeatable, interpretable, intuitive, human-understandable explanations. Factsheet, checklists, and technical specification requirements. Attention Visualization Documentation on visualizing and interpreting deep learning models.	Assess AI ethical principles via a maturity model methodology from a holistic perspective of people, process, and technology. Established proven boundary conditions that define a decision manifold and envelope and can be tested in a repeatable way. An XAI metric of evaluation proposed by Rosenfield (2021) suggests: “Four such metrics based on performance differences, D, between the explanation’s logic and the agent’s actual performance, the number of rules, R, outputted by the explanation, the number of features, F, used to generate that explanation, and the stability, S, of the explanation” [27]. Additional metrics include: Feature Importance Analysis LIME (Local Interpretable Model-Agnostic Explanations) Counterfactual Analysis Model Distillation

#### 2.2.2. Data Privacy

Data privacy (see Table 2) is of paramount importance for ethical AI as it safeguards individuals’ rights, autonomy, and personal information [28]. Ethical considerations in data privacy involve respecting privacy laws and regulations, obtaining informed consent, and implementing robust security measures to protect sensitive data [29]. Respecting privacy principles ensures that personally identifiable information is handled responsibly and that individuals have control over how their data are collected, used, and shared. By prioritizing data privacy, AI systems can foster trust, maintain confidentiality, and mitigate potential risks associated with unauthorized access or misuse of data. Protecting data privacy is essential for upholding ethical standards and ensuring the responsible development and deployment of AI technologies [30,31]. Improving trust in AI requires improved metrics for evaluating data privacy in AI, including but not limited to:

**Data Minimization:** Assessing the extent to which AI systems minimize the collection and retention of personally identifiable information (PII) to reduce privacy risks [32,33].

**Anonymization and De-identification:** Evaluating the effectiveness of techniques used to anonymize or de-identify personal data, ensuring that individuals cannot be re-identified from the data [34,35].

**Access Controls and Encryption:** Assessing the implementation of access controls and encryption mechanisms to protect sensitive data from unauthorized access or disclosure [36]. 

**Privacy Impact Assessments:** Conducting privacy impact assessments to identify and address privacy risks associated with AI systems, including data collection, processing, and storage [37].

**Transparency and User Consent:** Evaluating the transparency of data practices and the effectiveness of obtaining informed user consent for data collection and processing activities [38].

Preserving data security and privacy is vital in the age of Large Language Models (LLMs) and data-driven services. Personally Identifiable Information (PII) varies by country as a legal, not technical, concept. In the US, PII is information revealing identity, while the EU’s “personal data” term, governed by the General Data Protection Regulation (GDPR), includes identifiers like IP addresses and extends across various categories. The spotlight on private data as a product, not just a free app, underscores the demand for stronger ethical, moral, and legal privacy measures in AI applications. In the absence of these guardrails, we might be creating a world fraught with bias, disinformation, security, privacy, and legal challenges, including, but not limited to:

**IP Theft:** Samsung recently prohibited its staff from using ChatGPT after a recent data breach occurred. Significant concerns regarding the potential leakage of sensitive user data collected by LLMs is leading other organizations to curtail their use.

**Disinformation and Bias:** LLMs have gained a reputation for disseminating inaccurate or biased information, raising concerns among governments about the possibility of malicious actors employing LLMs to propagate propaganda.

**Legal Issues:** The issue of copyright infringement in relation to AI has surfaced, encompassing various aspects such as unauthorized usage of cover art and the creation of counterfeit songs attributed to artists like Drake. This matter has also become a significant factor in the recent strike by the Writers Guild of America (WGA), with writers expressing concerns that studios could generate AI-generated stories without their involvement.

**Education:** Teachers share apprehensions as students increasingly rely on LLMs for their homework. This concern was highlighted when Chegg, an educational technology company, observed a significant increase in students turning to ChatGPT, resulting in a more than 40% drop in Chegg’s stock value in one week.

**Economy:** The issue of job security is causing anxiety in relation to artificial intelligence. Recently, IBM halted the hiring process for approximately 8000 positions that it believed could be substituted by AI. Furthermore, IBM indicated that AI has the potential to replace almost one-third of its non-customer-facing positions. According to certain projections, around 300 million jobs could be influenced by AI, although Goldman Sachs estimates that AI could also contribute to a 7% increase in global GDP [39].

A ChatGPT query on how it is protecting data privacy or more specifically to “provide examples of proprietary data that have been fed to chat-GPT” notes: “As an AI language model, I don’t have direct access to real-time data or specific information about proprietary data fed to ChatGPT” [40]. Similar to the Amazon Web Services shared security model, ChatGPT notes: “the responsibility to ensure appropriate data handling practices also lies with the users and organizations interacting with the model.” Moreover, OpenAI, the organization behind ChatGPT, takes data privacy seriously and implements various measures to protect user data, including:

**Data anonymization:** Precautions to remove personally identifiable information (PII) from the training data used for language models. This helps to ensure that specific individuals cannot be identified through the generated responses.

**User data storage:** Does not store user-specific data beyond the duration of the conversation. Once the conversation is completed, user inputs are typically discarded and not used for further training or analysis.

**Security measures:** Maintains robust security protocols to protect the data it handles. This includes employing encryption, access controls, and monitoring to safeguard against unauthorized access or data breaches.

**Compliance with regulations:** Strives to comply with relevant data protection and privacy regulations, such as the General Data Protection Regulation (GDPR) in the European Union, which aim to provide transparency and control to users regarding their data [40].

Despite these privacy safeguards, Apple, Samsung, and other large companies have banned or limited use of LLMs due to the risk of proprietary data being used. A ChatGPT query noted that some examples of proprietary data that may be inputted include:

**Customer or user data:** This can involve anonymized or aggregated data collected from user interactions, such as chat logs, customer support conversations, or user feedback.

**Company-specific knowledge:** Proprietary information or expertise related to a particular industry, domain, or organization that can be used to enhance the model’s understanding and generate more contextually relevant responses.

**Research and development data:** Data from internal research, experimentation, or development processes, which may include prototypes, trial data, or proprietary algorithms.

**Intellectual property:** Confidential information, trade secrets, or patented algorithms that are used to train the model and provide unique capabilities or competitive advantage.

**Partnerships and collaborations:** Data shared under specific agreements or collaborations with external partners, which can include data from joint research projects, shared resources, or cross-organization datasets [40].

It is important to note that the specifics of proprietary data used for training AI models like ChatGPT may vary depending on the organization, data sharing agreements, and data protection policies in place. As the adoption, reliance, and value of GAIs and LLMs increase, it is critical to continuously improve the security and privacy guardrails to ensure these gains are sustainable. This requires improvements in the metrics of evaluation to measure the effectiveness of related privacy processes, policies, and technology.

#### 2.2.3. Technical Robustness and Safety

**Applying a lens of technical robustness and safety to improve methods and analysis of trust in AI.**

Measuring technical robustness and safety in AI systems involves assessing various metrics and evaluation criteria (see Table 3). It is important to note that the specific metrics and evaluation criteria may vary depending on the application domain, system complexity, and the level of safety and robustness required. Evaluation frameworks like the NIST AI Metrics Suite, AI System Safety Framework, or industry-specific guidelines can provide further guidance in measuring technical robustness and safety in AI systems [41]. Some commonly used metrics for evaluating these aspects include:

**Adversarial Robustness:** This metric evaluates the system’s resilience against adversarial attacks, where intentional perturbations or manipulations of input data are designed to mislead or deceive the AI system [42].

**Generalization Performance:** Generalization measures the system’s ability to perform well on unseen or out-of-distribution data. Metrics like accuracy, precision, recall, or F1 score on validation or test sets are commonly used to assess how well the AI system generalizes its learned knowledge to new instances [43].

**Stability and Sensitivity Analysis:** Stability refers to the consistency of an AI system’s output when subjected to variations in input or environmental conditions. Sensitivity analysis measures the extent to which changes in input data affect the system’s output. These analyses help evaluate the system’s reliability and consistency [44].

**Error Analysis:** Examining the types and patterns of errors made by the AI system can provide insights into its limitations and potential safety risks. Identifying the types of errors, such as false positives, false negatives, or bias in predictions, helps users to understand and mitigate potential harm or biases in decision-making [45].

**Coverage and Edge Cases:** Evaluating the system’s performance on a diverse range of inputs, including edge cases and corner cases, is essential to understand its limitations and potential failure modes. Metrics like coverage of input space, performance on rare or critical events, or performance in extreme conditions can be used [46].

**Safety Constraints and Compliance:** Assessing whether the AI system adheres to safety constraints, regulations, and compliance standards is crucial. Compliance with ethical guidelines, legal requirements, and industry-specific safety standards ensures that the system operates within defined boundaries and mitigates potential risks [47].

**Failure Modes and Risk Analysis:** Conducting comprehensive risk analysis to identify potential failure modes and associated risks is important. This involves evaluating the severity, likelihood, and potential impact of system failures or errors in different scenarios [48,49].

**Table 3:** Technical Robustness and Safety documentation and metrics of evaluation in AI-TMM [50].

Key AI Ethical Requirements—Trust Factors and Assumptions	Documentation	Metric of Evaluation
**Trust Factors and Assumptions:** Resilient to attacks on confidentiality, integrity, and availability. Redundancy, agility, and response plan is documented and tested. Accuracy levels high and reproducible. Reliable and explainable results.	Penetration testing results and maturity level assessment. Response to all hazards tested and assessed against reproducibility checklists. Documentation of accuracy under adversarial examples (e.g., using the L∞ norm), robustness against known attacks, or certification techniques like robustness verification can be employed.	Metrics of evaluation for AI safety include but are not limited to: Likelihood of the AI causing harm or unintended consequences in its decision-making or actions. Another metric could be the AI’s ability to align its goals with those of human stakeholders, or the robustness of the AI’s decision-making process to errors or malicious attacks. Metrics for evaluating technical robustness and safety in AI systems include adversarial robustness, generalization performance, stability analysis, error analysis, coverage of edge cases, compliance with safety constraints, and risk analysis.

#### 2.2.4. Transparency

Transparency (see Table 4) plays a vital role in ensuring the ethicality of AI by fostering accountability, trust, and the capacity to address potential biases and unintended consequences [51]. Through the provision of transparent documentation, disclosure of the data sources and algorithms employed, and facilitation of external review, transparency enables the assessment of fairness, reliability, and potential risks associated with AI systems. It empowers stakeholders to comprehend the decision-making processes of AI and identify and rectify any biases or errors that may emerge. Furthermore, transparency promotes responsible deployment of AI, instilling public confidence and facilitating well-informed decision-making [52]. Metrics for evaluating AI transparency focus on the interpretability and explainability of AI systems, and include:

**Explainability Methods:** Evaluating the effectiveness of different explainability techniques, such as feature importance analysis, rule-based explanations, or model-agnostic methods like LIME or SHAP, to understand how well the AI system can provide interpretable explanations for its decisions [20,53].

**Model Complexity:** Assessing the complexity or entropy of the AI model and its impact on transparency. This involves measuring the number of parameters, layers, or the overall architecture’s interpretability to determine the degree to which the model can be understood by humans [21,54].

**Intelligibility of Output:** Evaluating how well the output of the AI system is understood by end-users or stakeholders. This can involve measuring the clarity, comprehensibility, and usefulness of the provided information or predictions to ensure transparency in the decision-making process [55].

**Documentation and Annotations:** Assessing the availability and quality of documentation or annotations that accompany the AI system. This includes clear descriptions of the training data, model architecture, and assumptions made during the development process to enhance transparency [56,57].

**User Feedback:** Gathering feedback from users or stakeholders to assess their perception of the system’s transparency. This can involve surveys, user studies, or qualitative interviews to gauge their understanding of the system’s functioning and the explanations provided [58].

These metrics aim to quantify and evaluate the transparency of AI systems, allowing for better understanding and trust in the decision-making processes of these complex models. These approaches can build on existing AI transparency research, including but not limited to: “Saliency maps [59], self-attention patterns [60], influence functions [61], probing [62], i.e., counterfactual [63], contrastive [64], free text [65], concept-level explanations [66]”.

#### 2.2.5. Data Use and Design

Data Use and Design play a critical role in upholding the ethical standards of AI by shaping the fairness, accuracy, and potential biases present in AI systems [67]. Ethical considerations in data usage encompass the careful selection and preparation of datasets that are inclusive, varied, and free from discriminatory biases. Thoughtful design practices ensure that AI models are trained on dependable and pertinent data, preventing the perpetuation of unjust or detrimental results. By adhering to ethical principles during data collection, preprocessing, and model training, AI systems can mitigate biases, promote fairness, and ensure equitable benefits for all individuals and communities [54,68]. Metrics for evaluating data use and design in AI systems focus on responsible data practices, and design considerations are critical for improving trust in generative AI, LLMs, and other AI innovation. These metrics include but are not limited to:

**Data Privacy Compliance:** Assessing the system’s adherence to data privacy regulations and best practices, such as General Data Protection Regulation (GPDR) and the Health Insurance Portability and Accountability Act (HIPAA) to ensure proper handling, storage, and privacy protection of user data [69].

**Data Bias Analysis:** Evaluating the presence of biases in training data and the resulting impact on the system’s outputs. This involves measuring disparate impact, fairness, or demographic parity to address potential biases in decision-making [70].

**Data Governance:** Assessing the establishment and implementation of robust data governance policies and frameworks within organizations. This includes metrics related to data quality, data provenance, and data management practices to ensure responsible data use [71].

**Ethical Data Sourcing:** Evaluating the ethical considerations in data sourcing, such as obtaining consent, ensuring data diversity, and avoiding unethical data acquisition practices, to promote fairness and avoid potential harm [72].

**Human-in-the-Loop Evaluation:** Incorporating human feedback and evaluation in the AI system’s design and iterative development process. This involves metrics like user satisfaction, usability, and user-centered design principles to ensure human-centric and ethically aligned AI systems [9].

Ethical AI data use and design (see Table 5) is a critical component of the proposed AI Trust Framework. This includes ethical practices in the AI data lifecycle, taking into account the potential societal impact and safeguarding individuals’ rights and privacy. This involves promoting transparency, fairness, accountability, and obtaining informed consent throughout the development of AI algorithms and models. Ethical considerations extend to all stages of data management, including collection, storage, processing, and sharing, to ensure that the development and deployment of AI technologies align with ethical principles and legal requirements. Floridi and Cowls (2019) advocate for the integration of ethical considerations early in the development process, such as identifying and addressing biases in training data and algorithmic decision-making; they also emphasize the need for explainability and accountability mechanisms in AI systems to enable users to comprehend decision-making processes and address potential harms [73]. Similarly, Mittelstadt et al. (2016) highlight the significance of addressing privacy concerns in AI data use by advocating for clear and transparent privacy policies and data governance frameworks that protect personal information while permitting legitimate data use in AI applications. Achieving ethical AI data use and design necessitates collaboration among computer scientists, ethicists, legal experts, and policymakers, employing a multidisciplinary approach to ensure adherence to ethical standards and the protection of individuals’ rights and well-being [72].

#### 2.2.6. Societal Well Being

Considering societal well-being (see Table 6) in designing and managing AI technologies involves incorporating ethical considerations, addressing biases, promoting inclusivity, ensuring user safety, and fostering accountability. Recent calls from lawmakers, technologists, and concerned citizens have highlighted that societal well-being may be compromised if ethical guardrails are not included in AI advances [39]. This includes the potential missed opportunity for LLMs—like Chat GPT—to significantly improve the quality of life for society and its end users. Metrics of evaluation for AI societal well-being can include:

**Ethical Guidelines:** Implementing ethical guidelines that govern the behavior and content to prevent the generation of harmful or unethical content [74,75].

**Bias Mitigation:** Taking measures to identify and mitigate biases in responses, such as by improving the training data quality and implementing fairness-aware algorithms [57,70]

**Inclusivity and Diversity:** Ensuring that AI systems understand and respect diverse perspectives, cultures, and identities, and actively avoid generating content that promotes discrimination or exclusion [76].

**User Safety Measures:** Implementing safeguards to protect users from harmful content and misinformation, including content moderation mechanisms, detection of harmful behavior, and providing appropriate warnings or disclaimers [77,78]

**Accountability and Transparency:** Promoting transparency by providing clear information about the capabilities and limitations of AI systems, disclosing the data sources used, and enabling external auditing to ensure accountability [79,80].

Additional pillars that underpin societal wellbeing may also include:

**Fairness:** evaluating bias and equity for various background or demographic characteristics. 

**Inclusivity:** ability to accommodate and serve the needs of a diverse population.

**Privacy:** measuring the AI system’s compliance with regulations and best practices for protecting individuals’ personal information.

**Transparency:** ensuring that the AI system’s decision-making process can be understood and explained by humans.

**Accountability:** assessing the AI system’s ability to be held responsible for its actions, and the processes in place for addressing negative impacts.

**Human autonomy:** measuring the degree to which the AI system respects and preserves human agency and decision-making.

**Economic impact:** evaluating how the AI system affects the job market and overall economy.

**Environmental impact:** assessing how the AI system affects the natural environment and its sustainability.

#### 2.2.7. Accountability

When an AI system does not perform as it was designed, trust is lost. Who is held accountable when an LLM or AI bot developed the code that led to a failure? Improving accountability is critical to improving trust in AI. Accountability helps to ensure that those involved in the development, deployment, and utilization of AI systems take ownership of their actions and the outcomes produced by these systems. This responsibility encompasses the decision-making processes and results generated by AI algorithms. Accountability necessitates clear delineation of responsibilities, transparency in the development of AI technologies, and the implementation of mechanisms to address biases, errors, and unintended consequences. By upholding accountability, individuals, organizations, and institutions are held answerable for their AI systems, thereby upholding principles of fairness, transparency, and user protection. This fosters trust among users, stakeholders, and the wider public, promoting ethical practices and mitigating potential harm [51,72,81].

The following can help improve accountability and trust in AI (see Table 7):

**Model Performance Monitoring:** Involves continuously observing the performance of AI models over time to guarantee compliance with predefined performance criteria and standards [82,83].

**Bias Detection and Mitigation:** Helps improve accountability by implementing techniques to identify and address biases within AI systems, ensuring impartial and equitable outcomes [84,85].

**Explainability and Interpretability:** Focuses on evaluating the degree to which AI systems provide explanations or justifications for their decisions and actions, enabling stakeholders to comprehend the rationale behind the generated outcomes [22,52].

**Transparency and Auditing:** Aims to enhance transparency by offering clear documentation, disclosing information about the training data and algorithms used, and facilitating external audits to ensure accountability [51,86].

**Error Analysis and Feedback Mechanisms:** Involve conducting comprehensive error analysis, soliciting user feedback, and implementing mechanisms to learn from mistakes and enhance the performance of the AI system [53,87].

## 3. Method

Harnessing the power of AI in a sustainable and ethical way will require significant improvements in trust. This following case study applies the AI Trust Framework to examine security, privacy, and ethical requirements for training and deploying AI systems. While the following study is illustrative, it is completely plausible and timely as companies—like Apple Inc—join the ranks of companies, including Amazon, Samsung, and JP Morgan Chase, in prohibiting some employees access to ChatGPT and similar AI platforms. Apple’s decision stems from concerns that the utilization of such programs by employees may lead to the disclosure of sensitive information [88].

The AI-TMM methodology is applied to two different use cases below with different levels of trust as defined by the seven pillars. For the maturity indicator level (MIL) scoring, the use case focuses on the maturity level of AI explainability. Explainable AI (XAI) is one of the seven critical pillars of the AI-TMM. While applying a MIL score for each of seven pillars is beyond the scope of this study, it should be applied for a holistic application of the AI-TMM. The maturity model methodology is easy to follow, providing a modular and repeatable framework to measure documentation, management, and testing. The following use case incorporates the three critical elements that make up the AI-TMM:

***The AI Trust Seven pillars (***Table 1, Table 2, Table 3, Table 4, Table 5, Table 6 and Table 7***):*** Explainability (XAI), ii. Data privacy, iii. Robustness and Safety, iv. Transparency, v. Data Use and Design, vi. Societal Well-Being, vii. Accountability.

***The Maturity Indicator Level (MIL)*** *scoring for* measurement and evaluation of critical documentation, management, and testing of AI systems:

- Fully Implemented (MIL Score of 3),
- Largely Implemented (MIL Score of 2),
- Partially Implemented (MIL Score of 1),
- No control (MIL Score of 0).

***Implementation Process*** for continuous and repeatable assessment (See Figure 1).

Step 1: Determine Governing Frameworks and Controls

Step 2: Perform Assessment

Step 3: Determine and Analyze Gaps

Step 4: Plan and Prioritize: 

Step 5: Implement Plans:

## 4. Results

**The results highlight an illustrative use case which provides realistic examples of the AI-TMM Seven pillars (**Table 1, Table 2, Table 3, Table 4, Table 5, Table 6 and Table 7**):** A top software engineer at Apple Inc. used an LLM platform with poor secure software development lifecycle documentation (Table 1). The engineer appreciated her improved time to value in developing with help from the LLM that produced a python script for anomaly detection. That code was included in a new security application designed to alert users that their PII was being used by an LLM bot. The lack of documentation was exacerbated by poor data access controls. This created a lack of awareness, and unbeknownst to the engineer, the code was previously introduced to the LLM via a large data dump by hackers that targeted a competing company. When the application was launched, it led to a surprising claim by this competitor of exposing the company’s PII (Table 2). Subsequently, in an offline test, the data breach by the Apple engineer could not be replicated via testing (Table 3), reducing trust in the AI software. Anomaly detection logs were searched but found to be inadequate (Table 4). Moreover, in the replay, bias was uncovered (Table 5). Apple leadership requested that a highly regarded technical consultant be hired to provide an in-depth audit of the AI software, the company itself, and to provide immediate steps that can be adopted quickly to limit the adverse impacts of the privacy breach (Table 6). One major area of improvement was adding a secure software development lifecycle that controlled data access and lineage through the lifecycle (Table 7).

Use Case: Lack of Trust in AI due to Lack of XAI Maturity: Table 8 below highlights gaps found in the use case with low levels of XAI maturity as measured by the AI-TMM methodology.

The use case applied the AI-TMM and found a low level of maturity for XAI controls highlighted in Table 9 below. The findings highlighted that there were no XAI controls that were documented, managed, or tested. Thus, a low maturity indicator level of 0 was assigned to each category. This led to detrimental entropy production in a company that abandoned some use of AI because it lacked explainability in design and management of AI systems. This case study is exemplary of Apple Inc’s recent blocking of Chat GPT use by some employees.

**Control Use Case Highlighting High Levels of XAI:** The AI control use case highlights an AI system where there is a high level of AI explainability as defined by XAI requirements in Table 1 and as measured by the AI-TMM in the design and management of an AI system. This use case highlights how application of the AI-TMM via a maturity model approach may help improve the trust score as defined by XAI requirements and AI-TMM maturity indicator level measures. The illustrative output shows how Apple and other industry leaders can incorporate XAI in the design and management of their AI systems to enable secure internal use of AI as well as produce more trustworthy AI products.

Finally, following the AI-TMM methodology, baseline assessment, gap analysis, and mitigation plans were prioritized adding controls from the seven pillars (Table 1, Table 2, Table 3, Table 4, Table 5, Table 6 and Table 7), helping Apple’s AI users and its customers to regain trust.

Cybersecurity regulations, as outlined in our Introduction, do not inherently establish order on their own. Compliance does not equal security. A defense in depth or zero trust approach from companies is an ongoing necessity. At times, these cyber regulations can appear arbitrary, formulated to exert control over a firm, yet they remain obligatory for companies in that nation, as seen with Didi in China [89]. In the United States, regulations are formulated not only to safeguard firms but also to protect U.S. interests, including military aspects [90]. The advent of deep learning (DL) has enabled firms to design rules to more efficiently safeguard prodigious data sets by improving inference and fidelity of data insight. For example, models can be trained to automatically classify data into different categories based on its sensitivity. They can identify personally identifiable information (PII), financial data, health records, or other regulated data types. This helps in ensuring that sensitive data is properly handled and protected according to regulations. DL refers to a collection of multi-layered machine learning algorithms proficient in extracting high-level abstractions from vast, complex datasets. These algorithms often acquire feature representations through numerous nonlinear hidden layers, automating feature engineering [91].

However, the recent introduction of ChatGPT has elevated the significance of cybersecurity, as hackers employ strategies that make detecting cyber-attacks even more challenging. According to a report in the Wall Street Journal, consumers should exercise caution: AI chatbots like ChatGPT are poised to amplify the utilization and effectiveness of online fraudulent tools such as phishing and spear-phishing messages [92]. Global instances of phishing attacks surged by almost 50% in 2022 compared to the previous year, as reported by Zscaler, a provider of cloud security. Artificial intelligence software that lends credibility to phishing messages exacerbates the issue. AI diminishes language barriers and grammatical errors, assisting scammers in impersonating a target’s associates, acquaintances, or family members [92].

These concerns assume significance not only for industries, enterprises, and governments, but also for consumers and users of medical devices. Medical devices must ensure the delivery of vital functions even when faced with adverse circumstances [93]. Importantly, Riegler and collaborators [93] echo the concern initially raised by Gartner [94]: By 2025, cyber attackers will have weaponized operational technology environments to inflict harm or fatality on humans. In 2021, Gartner had observed: Attacks on operational technology (OT)—encompassing hardware and software that oversee or control equipment, assets, and processes—have become more frequent. These attacks have evolved beyond immediate process disruption, extending to compromising the integrity of industrial control systems with the intent to cause physical harm. 

## 5. Discussion of the Case Study

In the illustrative use case shared, inadvertently copying and exposing a competitor’s proprietary code may have put Apple in jeopardy for legal liability, wasting its currently available free energy, sacrificing the availability of future free energy, and reducing the absolute maximum entropy that the corporation could produce. The accidental adoption of a competitor’s stolen code meant that Apple’s structural entropy production was unacceptably high, wasting free energy, and making it difficult to be productive. A technical expert apply the AI-TMM to identity and mitigate security and trust gaps in its AI software development lifecycle. As a result, Apple’s structural entropy production has been significantly reduced, providing more free energy (resources) to be applied to improve its productivity (maximum entropy production), to stabilize the business, and thereby to satisfy users and customers in trusting the revised AI software.

While trust is important, it is not a “be all and end all.” For example, the first computer “worm” used to infect numerous networked computers throughout the U.S. was released into the “wild” in interlocking networks where researchers were working on large machines that shared resources across a community that “operated largely on trust and prized availability of information over confidentiality and integrity” [95]. Almost immediately, the load caused by copies of the worm crashed networks across the U.S., including for military users, and networks at MIT and at RAND.

## 6. Conclusions

The AI-TMM is timely in providing an intuitive, modular and repeatable methodology and framework to bolster ethical AI guardrails. Geoffrey Hinton, also known as the “Godfather of AI”, recently quit his position at Google after deciding he had to “blow the whistle” on the technology he helped develop [96]. In absence of these ethical guardrails, Hinton warns: “It’s very possible that humanity is just a phase in the progress of intelligence. Biological intelligence could give way to digital intelligence. After that, we’re not needed. Digital intelligence is immortal, as long as its stored somewhere” [96]. As AI innovation and adoption grows exponentially, it is imperative to improve the metrics for evaluating, developing, and managing trust in AI systems. This is especially when AI is applied to high assurance critical infrastructure environments (e.g., defense, energy, healthcare, transportation, etc.) where challenges with transparency, uncertainty, conflict, and competition are exacerbated when trust is lost [10].

To help overcome these challenges, an entropy lens can be applied to help improve how we measure, design, and manage trust in AI in complex environments. This can also help us build systems that are able to adapt to a wide range of complex envriomenst and perturbations, while also ensuring that the system remains within certain constraints or boundaries [2]. This approach can help to minimize the risks of catastrophic failure and ensure that AI systems operate effectively and reliably in the face of uncertainty and unexpected changes [97]. These findings also suggest that the seven pillars of the proposed AI-TMM are critical attributes in enabling systems to function securely, ethically, and sustainably. As highlighted by the use case above, these attributes may help enable AI systems to maintain performance even in the face of unexpected inputs or disturbances. Future research should explore and validate effective application of the framework to other use cases involving critical infrastructures and high assurance systems that require 24x7 availability. Effective application of the AI-TMM will require careful consideration of the constraints and conditions under which the system will operate. This research would benefit from testing and validation of the system under a variety of scenarios to ensure that it can perform reliably under a range of conditions. In this context, the application of an entropy lens may prove valuable in building AI systems that can operate effectively and reliably in the face of uncertainty and unexpected changes.

Findings from this research highlight how entropy can be applied to improve ethical guardrails for AI applications operating in complex environments. For AI advances to to be incorporated into civilization in a sustainable way, intelligent algorithms and machines require trust. Future studies should examine opportunities to overcome tradoffs in ethical AI design and management, such as security versus efficiency, privacy versus explainability. Entropy will play a critical role in that exploration. For example, consider privacy preserving machine learning solutions that leverage Multi-Party Computation algorithms that require entropy for separate keys or shards, fragments of keys to be unpredictable, secure, and coordinated during generation and deployment. If the ML algorithms lack transparency in how they are generating their keys, it can be difficult to measure the level of the entropy to indicate effectiveness. Future research should focus on overcoming these tradeoffs by applying the ethical AI framework throughout the lifecycle of these systems.

Ethical AI is of paramount importance in the design and management of AI systems. The AI-TMM applied through an entropy lens helped provide a moral compass guiding the development and deployment of AI technologies. Ethical AI ensures that these systems are designed to include security best practices, explainbility, transparency, accountability, and privacy. Prioritizing ethical considerations help can mitigate the risks of biased algorithms, discriminatory outcomes, and unintended consequences. Moreover, ethical AI fosters trust among users and stakeholders, which is essential for widespread adoption. As AI becomes increasingly integrated into various aspects of our lives, from healthcare to finance and beyond, improved metrics of evaluation via AI-TMM may help uphold our values and principles but also safeguards against the potential misuse and harm that unchecked AI systems could bring. It is, therefore, incumbent upon developers, organizations, and policymakers to explore AI-TMM and other ethical AI frameworks as an integral part of responsible AI innovation and governance.

## Figures and Tables

**Table 2:** Data privacy documentation and metrics of evaluation in an AI Trust Framework.

Key AI Ethical Requirements—Trust Factors and Assumptions	Documentation	Metric of Evaluation (ME)
Trust Factors and Assumptions: Privacy is protected without losses in XAI that reduce repeatable, interpretable, intuitive, human-understandable explanations.	Technical assurances that autonomous systems and/or platforms (1) cannot steal or misuse data supplied and maintained by users; and (2) cannot access any sensitive data or IP to query, store, or use the model for any other than the contracted purpose. Impact Assessments Access control list ISO/IEC. (2013) ISO/IEC 27001:2013 Article 13 of the GDPR	Quantitative measures: These measures use statistical methods to estimate the amount of privacy a system provides. Examples include: Data Minimization, Anonymization and De-identification, Access Controls and Encryption, Privacy Impact Assessments, Transparency and User Consent, Evaluating the transparency of data practices and the effectiveness of obtaining informed user consent for data collection and processing activities. Qualitative measures: These measures are based on expert judgment or heuristics and are used to determine the level of privacy a system provides. User studies: These measures involve conducting experiments with real users to determine their perception of privacy in a system. Legal frameworks: Laws and regulations that specify what is considered private and how personal data can be collected, used, and shared. Ethical principles: Respect for autonomy or non-maleficence when evaluating the privacy of a system.

**Table 4:** Transparency documentation and metrics of evaluation in the AI-TMM.

Key AI Ethical Requirements—Trust Factors and Assumptions	Documentation	Metric of Evaluation
Transparency Trust Factors and Assumptions: Formal methods enable explainability. Adaptable user-centered explainability. Identify human and machine inputs and outputs to classify data transactions.	Checklist and Factsheets (explainability) Documentation on training data Saliency maps	A metric of evaluation for AI transparency could be the degree to which an AI system’s decision-making process can be understood and explained by humans. Explainability techniques, such as feature importance analysis, rule-based explanations, or model-agnostic methods like LIME or SHAP can be used to understand how well the AI system can be used. Level of access provided to the training data, model architecture, and decision-making logic of the AI system. Model interpretability and feature attribution that can be used to evaluate transparency of AI systems. Completely, accurately and clearly quantify the agent’s logic, refered to as transparency [26] and fidelity [22,27]. Heuristic documentation on normal as well as defined boundary conditions. Testing for false positives and negatives and other anomalies to better understand level of accuracy for the detection of anomalies.

**Table 5:** Data use and Design documentation and metrics of evaluation in the AI-TMM.

Key AI Ethical Requirements—Trust Factors and Assumptions	Documentation	Metric of Evaluation
AI Ethics in Data use and Design Trust Factors and Assumptions: Document, define and limit bias. Use diverse training data that optimize accessibility and universal design. Include human feedback loop.	AI Ethics checklists included through design and implementation of system lifecycle	Data Bias Analysis: Bias testing and defining classifiers and boundary conditions throughout ML algorithm training, learning, and implementation lifecycle. Data Governance Assess AI ethical principles via a maturity model methodology from a holistic perspective of people, process, and technology. Data Privacy Compliance Ethical Data Sourcing

**Table 6:** Societal Well-being documentation and metrics of evaluation in the AI-TMM.

Key AI Ethical Requirements—Trust Factors and Assumptions	Documentation	Metric of Evaluation
Societal well-being Trust Factors and Assumptions: Assess and limit adverse impact on individuals, groups, and society.	AI Ethics checklists and constraints on algorithms that could potentially cause societal detriments. Documentation of ethics, bias mitigation, user safety measures, etc. Data design and training documentation on accountability and transparency.	Metrics of evaluation for AI societal well-being can include: Ethical Guidelines, Bias Mitigation, Inclusivity and Diversity, User Safety Measures, Accountability and Transparency

**Table 7:** Accountability documentation and metrics of evaluation in the AI-TMM.

Key AI Ethical Requirements—Trust Factors and Assumptions	Documentation	Metric of Evaluation
Trust Factors and Assumptions: Validation and verification of algorithms, data, and design through lifecycle from training to application. Examine and document bias, assumptions, trade-offs in accuracy versus speed, etc.	Factsheet, checklists, and technical specification requirements that can be audited and explained. Monitoring and logging of deviations from “normal” heuristic and boundary conditions and assumptions. Audibility that confirms data provenance and non-repudiation through project lifecycle from design, training, and implementation.	Assess AI ethical principles via a maturity model methodology from a holistic perspective of people, process, and technology. Model Performance Monitoring Bias Detection and Mitigation Explainability and Interpretability Transparency and Auditing Error Analysis and Feedback Mechanisms

**Table 8:** Use Case: Lack of Trust in AI due to Low Levels of XAI Maturity.

AI-TMM Goal	AI-TMM XAI Documentation	AI-TMM XAI Management	AI-TMM XAI Testing	AI-TMM Maturity Indicator Level Score Total
AI produces results that are repeatable, interpretable, intuitive, and human-understandable explanations.	No documentation in place on the quantification of accuracy of results that are repeatable, interpretable, intuitive, human-understandable explanations.	No human was in the loop managing XAI requirements via AI development lifecycle.	No testing of XAI validation principles such as: Feature Importance Analysis Counterfactual Analysis Model Distillation	No Documentation, Management, or Testing (MIL Score of 0)

**Table 9:** Use Case: Trust in AI Due to High Levels of XAI Maturity.

AI-TMM Goal	AI-TMM XAI Documentation	AI-TMM XAI Management	AI-TMM XAI Testing	AI-TMM Maturity Indicator Level Score Total
Robust documentation, management, and continuous testing of the AI combined with key XAI principals create a high level of XAI maturity and trust to enhance the transparency and comprehensibility of complex AI models and their decision-making processes for human users.	Robust documentation in place on the quantification of accuracy of results that are repeatable, interpretable, intuitive, human-understandable explanations.	Dedicated management of XAI requirements throughout the AI development lifecycle.	Continuous testing and XAI validation principles are incorporated throughout the AI lifecycle, including: Feature Importance Analysis, Counterfactual Analysis, and Model Distillation	Fully Implemented Documentation, Management, and Testing (MIL Score of 3)

## Data Availability

Data from findings and results is included in the publication and reprouducable by following the instructions of the AI-TMM.
